# Losses, inefficiencies and waste in the global food system

**DOI:** 10.1016/j.agsy.2017.01.014

**Published:** 2017-05

**Authors:** Peter Alexander, Calum Brown, Almut Arneth, John Finnigan, Dominic Moran, Mark D.A. Rounsevell

**Affiliations:** a School of Geosciences, University of Edinburgh, Drummond Street, Edinburgh EH8 9XP, UK; b Land Economy and Environment Research Group, SRUC, West Mains Road, Edinburgh EH9 3JG, UK; c Karlsruhe Institute of Technology, Institute of Meteorology and Climate Research, Atmospheric Environmental Research (IMK-IFU), Kreuzeckbahnstr. 19, 82467 Garmisch-Partenkirchen, Germany; d The Centre for Australian Weather and Climate Research – A partnership between CSIRO and the Bureau of Meteorology, CSIRO Marine and Atmospheric Research, Canberra, Australia; e Environment Department, University of York, University Rd, York YO10 5DD, UK

## Abstract

Losses at every stage in the food system influence the extent to which nutritional requirements of a growing global population can be sustainably met. Inefficiencies and losses in agricultural production and consumer behaviour all play a role. This paper aims to understand better the magnitude of different losses and to provide insights into how these influence overall food system efficiency. We take a systems view from primary production of agricultural biomass through to human food requirements and consumption. Quantities and losses over ten stages are calculated and compared in terms of dry mass, wet mass, protein and energy. The comparison reveals significant differences between these measurements, and the potential for wet mass figures used in previous studies to be misleading. The results suggest that due to cumulative losses, the proportion of global agricultural dry biomass consumed as food is just 6% (9.0% for energy and 7.6% for protein), and 24.8% of harvest biomass (31.9% for energy and 27.8% for protein). The highest rates of loss are associated with livestock production, although the largest absolute losses of biomass occur prior to harvest. Losses of harvested crops were also found to be substantial, with 44.0% of crop dry matter (36.9% of energy and 50.1% of protein) lost prior to human consumption. If human over-consumption, defined as food consumption in excess of nutritional requirements, is included as an additional inefficiency, 48.4% of harvested crops were found to be lost (53.2% of energy and 42.3% of protein). Over-eating was found to be at least as large a contributor to food system losses as consumer food waste. The findings suggest that influencing consumer behaviour, e.g. to eat less animal products, or to reduce per capita consumption closer to nutrient requirements, offer substantial potential to improve food security for the rising global population in a sustainable manner.

## Highlights

• Food system losses quantified from primary production to human food requirements.
• Global agricultural dry biomass consumed as food is 6% (energy 9.0% and protein 7.6%).
• 44% of harvested crops dry matter lost prior to human consumption.
• Highest loss rate in livestock production, but largest losses before harvest
• Over-eating at least as large a contributor to food system losses as consumer waste.

## Graphical abstract

**Figure:**
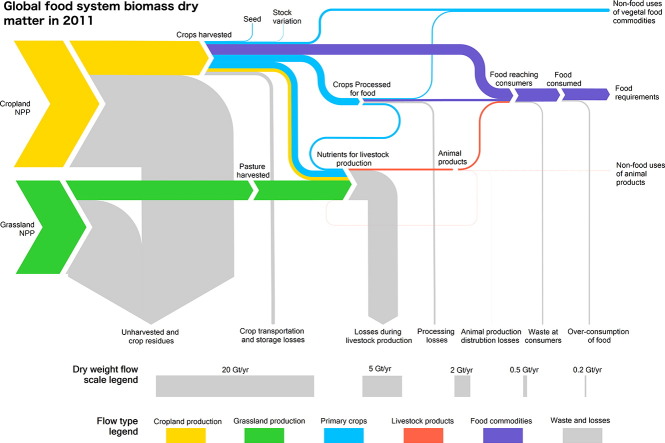

## 1 Introduction

The global food system is subject to the conflicting pressures of delivering the food demanded by an expanding and increasingly affluent population, while helping to achieve environmental sustainability (Godfray et al., 2010, Tilman and Clark, 2014). Along with rising population, higher consumption rates for commodities such as meat and milk, due to rising incomes (Kearney, 2010, Keyzer et al., 2005, Tilman et al., 2011), and increasing non-food demands for agricultural commodities, principally for bioenergy (Müller et al., 2008), all increase the pressures on agriculture. This situation is further complicated by climate impacts, leading to changes in land suitability and crop and animal yields (Müller and Robertson, 2014, Nelson et al., 2014). Meeting food demands either by expanding agricultural areas, causing land use change, or the intensification of production (i.e. seeking higher yields through the use of greater inputs, such as fertilisers, pesticides or water, or changes in management practices) have the potential to cause environmental harm, including greenhouse gas emissions (GHGs), deteriorating soil quality, use of scarce water and biodiversity loss (Cassman, 1999, Johnson et al., 2014, Smith et al., 2013). These impacts need to be reduced, particularly GHGs (currently 30% of all anthropogenic emissions (Le Quéré et al., 2015)) if international climate change targets are to be met (Benton and Bajželj, 2016).

Achieving greater food security in a sustainable manner requires improved food system efficiency. Production practices and consumer preferences, including diet and waste rates, influence the efficiency of the food system in producing agricultural biomass and its use in meeting human nutritional requirements (Smil, 2004). Approaches to achieving this objective have considered changes to agricultural production systems (Garnett et al., 2013, Smith, 2008, Tilman et al., 2011), the role of diet and the potential for demand side measures (Bajželj et al., 2014, Lamb et al., 2016, Smil, 2013, Stehfest et al., 2009), and the reduction of food waste (Gustavsson et al., 2011, Hall et al., 2009, Smith, 2013).

Although many studies have established that reducing food losses and waste may play a substantial role in achieving food security and climate change mitigation (Foley et al., 2011, Hall et al., 2009, Smith, 2013, West et al., 2014, WRAP, 2015), few have analysed the sources and distribution of global food losses and waste. The most highly cited study on food losses and waste to date, Gustavsson et al., (2011) calculated that approximately a third of food is lost or wasted from production to consumption, assuming loss rates for each region, process stage and commodity group, and applying these to the harvested quantities in FAO food balance data (FAOSTAT, 2015a). The study was based on a wide range of estimated and assumed loss rates (Gustavsson et al., 2013), making it problematic to check the validity of assumptions. Kummu et al. (2012) applied a similar approach (and loss rates) to calculate global food losses in energy terms to be 24%. These studies extend the work of Parfitt et al. (2010), which provided food losses for some countries/regions, but did not present global values. As a result, independent, comparable and transparent figures for food system losses are lacking. Further, losses occurring due to food consumption exceeding nutritional requirements have received even less attention, with limited research on consumption in the USA (Blair and Sobal, 2006, Eshel and Martin, 2006, Smil, 2004). There is also a gap in the understanding of the impact of livestock production on both food system biomass efficiency and feed crop losses.

This study provides a new, primarily empirically based assessment of losses in the food system as a whole. The sources of losses (from inefficiencies and waste) are considered from primary production of agricultural biomass through to the food required for human nutrition. The analysis improves the estimates of losses occurring through the food production-supply-consumption chain, and provides insights into system efficiency and the magnitude of losses at different stages. This clarifies the role of research into agricultural production (e.g. sustainable intensification) and consumer behaviours (e.g. related to diet and waste) in their wider food system context. A further aim is to explore the impact of calculating losses in the food system on the basis of different quantities or indicators (i.e. wet and dry mass, protein or energy). Finally, the work also makes greater use of available empirical data than previous studies for losses in the food system.

## 2 Method

### 2.1 Definitions and food system scope

This study considers losses to the food system at stages through production, supply and consumption. The variety of food system typologies and divergent production processes means that any characterisation of global system efficiency is liable to be contested. Although losses and inefficiencies are inevitable within any system, there is additionally a notional economic level of loss at which the implicit costs of altering the system to reduce losses outweighs the benefits in terms of avoided losses, e.g. perhaps due to the social or environmental impacts. It may be possible to explore the optimal level of food system losses given all externalities (where losses are also considered an externality), but this is highly challenging due to the complexity of the trade-offs, and the required valuation of associated non-market goods. However, such considerations are outside of the scope of this study, with its concern on understand and quantifying loss in the current global food system.

The food system definition used here includes biomass inedible by humans, e.g. by-products of food crop processing. Losses of inedible biomass are a source of inefficiency within the food system, increasing the environmental impacts of agriculture and reducing the quantity of food produced. The term ‘waste’ is used solely with regards to losses incurred by the consumer. The final use of commodities is considered, rather than the intended use. Therefore, if a commodity is intended for human consumption but is ultimately used for animal feed, perhaps as a result of spoiling or damage, this is accounted for as animal feed. This differs from previous work on food losses and waste (Gustavsson et al., 2011), which counted “unplanned” non-food uses as losses.

The ability of livestock to convert processing by-products into food has been argued to provide a useful service, delivering food from what might otherwise be waste material (Oltjen and Beckett, 1996, Sabiiti, 2011). This argument implicitly assumes that the same quantity of by-product would be produced, and not given another useful purpose, if it were not fed to animals. Excluding by-products when considering losses (e.g. (Gustavsson et al., 2011, Kummu et al., 2012)) implicitly follows a similar assumption. However, in both cases this assumption is questionable. For example, the value of commodities produced from the processing of oil crops is split relatively equally between oil and the ‘by-product’ meal (Alexander et al., 2016a). If the oil crop meals were not used for animal feed, the economic case for growing soybeans would be substantially altered, potentially leading to an alternative productive use for the meal (e.g. in bioenergy), or the substitution of some of the oil crop production with a more economically beneficial crop. Consequently, the use of such by-products should be ascribed some value when considering their impacts (Elferink et al., 2008).

### 2.2 Types of losses

Food system losses were considered in six categories, as follows:

***Agricultural production***: losses that occur in the production process. The losses include agricultural residues (e.g. roots and straw), unharvested crops and the losses during harvest.
***Livestock production:*** losses and inefficiencies in the conversion of feed and grass into animal products.
***Handling, storage and transportation:*** losses due to spillage and degradation during storage and distribution. These losses occur for primary crops, processed commodities and animal products.
***Processing:*** losses during the processing of commodities.
***Consumer waste:*** losses and waste between food reaching the consumer and being eaten.
**Over-consumption:** the additional food intake over that required for human nutrition (Blair and Sobal, 2006).

The loss or inefficiency types here cannot be directly classified as either wholly avoidable and unavoidable, as the production and processing types contain both elements in uncertain proportion. For example, the production of cereals necessarily involves the growing of roots and straw that form agricultural residues. Improved plant breeding or changes in management practices may increase the efficiency of cereal production, but there must be both practical and theoretical limits to these improvements. Furthermore, there are additional complexities in attempting to divide ascribe what losses are avoidable due to the connections across the food system, e.g. reductions in consumption has the potential to reduce losses that occur ‘unavoidably’ in production of that commodities.

### 2.3 Calculation of quantities and losses

**Fig. 1:**
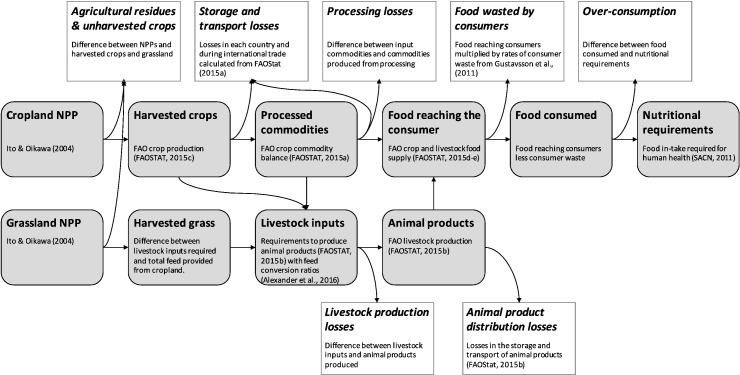
Food system stages associated losses, and summary of approaches used to estimate each quantity.

Fig. 1 shows the relationship between food system stages and associated losses. It also outlines the estimation method used for each value. Descriptions for each quantity (both total quantities and losses) are detailed below, with the order reflecting the calculation order. Each quantity was estimated in dry and wet mass, energy and protein terms. Values were calculated for 2011, as the most recent date for which all required data were available (FAOSTAT, 2015a, FAOSTAT, 2015b).

### 2.4 Cropland and grassland production

Global net primary production (NPP) has been the subject of much research (Del Grosso et al., 2008, Monfreda et al., 2008, New et al., 2009), but few studies provide NPP values disaggregated by land cover type. Global NPP values of 8.0 petagrams Carbon (PgC)/yr for cropland and 5.9 PgC/yr for grassland were used here (Ito and Oikawa, 2004), with cropland assigned from heath & moorland, warm or hot shrub & grassland, and Tibetan meadow/Siberian highland. The NPPs were converted to dry biomass by multiplying by a factor of 2, and then to energy, protein and wet mass by using calorific value, protein and moisture contents (adapted from SAC (2013) and Teagasc (2014) for grassland, and Krausmann et al. (2013) and Wirsenius (2007) for cropland). Table S1 shows the values used and the resulting NPPs for global cropland and grassland, in mass, energy and protein terms.

### 2.5 Harvested crop, processed commodities, animal product & food reaching the consumer

FAO production and commodity balance data were used to calculate quantities of harvested crops, processed commodities and food reaching the consumer (FAOSTAT, 2015a, FAOSTAT, 2015b, FAOSTAT, 2015c). These data are given in terms of wet mass, and were converted to energy, protein and dry matter (DM) using nutritional data for each commodity considered (Table S2). The energy and protein contents per mass for foods were derived from the global average in 2011 from the food supply data (FAOSTAT, 2015d, FAOSTAT, 2015e). In cases where a commodity had zero or minimal human consumption (e.g. oil crop meals), the energy and protein values were not available in the FAO food supply data, and these values were taken from INRA et al. (2016). The dry matter content values for commodities used primarily for food were obtained from the USDA (2015) nutrient database, and for feed commodities from INRA et al. (2016). Quantities of 91 commodities (see Table S2), representing 99.4% of global food consumption by calorific value were included in the analysis. The commodities comprise 50 primary crops (plus forage crops grown for livestock feed, e.g. alfalfa and forage maize) that are directly grown, 32 processed commodities derived from them, and 8 livestock products.

The total quantities of harvested crops were calculated by aggregating values for the 50 primary crops from the FAO crop production data in 2011 (FAOSTAT, 2015c). The use of all crops were determined through the commodity balance data (FAOSTAT, 2015a, FAOSTAT, 2015b), which identifies the quantities of food reaching the consumer, animal feed, inputs to further processing, other non-food related uses, seed, stock variation and waste. The primary crops and processed commodities used for food reaching the consumer, processing and non-food uses were calculated by aggregating these data. A small amount of animal products (< 0.1%) is categorised as being processed, and these were assumed to be used for food. Eggs hatched in poultry production (0.4% of animal products) were included in the feed category of livestock production inputs. The livestock commodity balance data after these adjustments was used to calculate the quantities of animal products for food and non-food uses.

### 2.6 Storage and transportation losses

The FAO definition of waste includes all losses between harvest and the consumer. These losses are recorded per country, but there are additional losses occurring during international trade. The commodity balance data contain the level of imports and exports, which allowed the international trade losses also to be calculated. For example, total wheat exports in in 2011 were 182.9 Mt, but imports were only 178.0 Mt, suggesting that 4.9 Mt were lost in transit. This is seen for many commodities and over time, e.g. wheat international trade losses varied between 3.2 and 6.5 Mt from 2000 to 2011, with a mean of 5.3 Mt. Tomatoes have the highest losses in international trade, with an average loss of 13.4% during the same period. The calculated storage and transport losses take national and international losses into account by summing the country losses figures and the calculated losses in international trade. For example, in the case of wheat in 2011, the total loss is calculated as 31.3 Mt (26.4 Mt aggregated national losses and 4.9 Mt international trade losses).

### 2.7 Livestock inputs, harvested grassland and livestock production losses

Direct data on the quantity of grass consumed by animals or harvested were not available, although quantities of feed supplied to animals was calculated through aggregation of commodity balance data (as above). Therefore, animal feed conversion ratios (expressed as ratios of DM of feed required to the wet mass of edible animal product (Macleod et al., 2013)) were used to calculate the total feed DM that would have been needed to produce all animal products. Feed conversion ratios from Alexander et al. (2016a) were used, and vary from 25 kg DM feed/kg edible mass for beef to 0.7 kg DM feed/kg edible mass for milk. Summing the calculated feed requirements for each animal product gives the total livestock inputs. The deficit between the feed requirements and feed provided from vegetal commodities was assumed to be provided from harvested grassland (either through grazing or hay/silage production), and converted into energy, protein and wet mass terms (using grass nutritional values, Table S1). The losses during livestock production were calculated as the difference between the inputs from feed and harvested grass, and the animal product outputs from the livestock food commodity balance (as described above).

### 2.8 Agricultural production inefficiencies and losses

The losses during agricultural production were calculated as the difference between the total NPP and the harvested quantity, for cropland and grassland respectively. For cropland, this loss represents all NPP that is not present within harvested crops, and encompasses all roots (except for harvested root crops) and straw, as well as crops spilled during harvesting or remaining unharvested. These are principally agricultural residues that will break down in the soil and provide nutrients for subsequent crops, but their production does create a level of inefficiency.

### 2.9 Food consumed and food wasted by consumers

The food wasted by consumers was determined using an approach and loss rates based on Gustavsson et al. (2011). Consumer waste percentages were used for 8 commodity groups (e.g. cereals, fruits, vegetables, and meat; Table S2) and 7 global regions (e.g. Europe, sub-Saharan Africa and Latin America, see Table S3). The consumer losses for each commodity and country were determined by applying the associated loss rate (Table S4) to the food reaching consumers for that country (FAOSTAT, 2015a, FAOSTAT, 2015b). These losses were then aggregated to provide an estimate of the global food wasted by consumers. The food remaining after accounting for the quantities wasted was assumed to have been consumed.

### 2.10 Nutritional requirements and over-consumption

Energy and protein requirements of 9.8 MJ/person/day (2342 kcal/person/day) and 52 g/day were assumed, respectively, with any excess intake attributed to over-consumption (Blair and Sobal, 2006). These are mean values that account for variation in requirements. Energy intake requirements vary by level of physical activity, age and gender. For instance, average energy requirements for the population of UK adult females and males are respectively 8.7 MJ/day (2079 kcal/day) and 10.9 MJ/day (2605 kcal/day) (SACN, 2011). The 9.8 MJ/person/day mean of these values used here is somewhat higher than the 2100 kcal/person/day (or less) energy intake used in some previous studies (Eshel and Martin, 2006, Kummu et al., 2012, Smil, 2004), but accord with others (e.g. Springmann et al. (2016) used 2200–2300 kcal/person/day), and is likely to exceed the intake needed to avoid hunger or malnutrition (WFP, 2016). The protein requirement of adult men and women depends on body mass, with 0.8 g/kg of body mass required per day (Institute of Medicine, 2005). Assuming an average body mass of 65 kg, 52 g/day of protein is the minimum safe limit. Given a global population of 7013 million people in 2011, a requirement for the world's population was taken as 25.1 EJ/year of energy and 133 Mt/years of protein.

### 2.11 Embodied quantities

Comparing the losses occurring between stages in the food system is problematic, due to the sequence of stages, the recirculating flows and non-food uses. For example, in a hypothetical sequence of three processes each with a 20% loss, 41% of the total losses occur in the first process while just 26% occur in the third process, due to the compounding of losses (Fig. S1). Therefore, to give an unbiased comparison of losses through the food system, ‘embodied’ quantities and losses were calculated by pro-rata allocation of losses to the other uses at each stage. The actual loss rates from subsequent stages were then applied to the increased quantities representing the embodied inputs, to calculate an embodied loss. The outcome is that the losses in later stages take into account the quantities lost during previous stages. The percentage of losses occurring at each stage is the embodied loss at that stage divided by the sum of all embodied losses. Using the stylised example above, the embodied loss rates give an unbiased representation, where an equal proportion of the total loss (i.e. one third) is associated with each process (Fig. S1).

## 3 Results

The net primary production, food required for human consumption, and 7 intermediate quantities in the food system were determined in wet and dry mass, energy and protein terms, including the losses at each stage (Table 1). The quantities and losses through the food system are shown in

**Table 1:** Mass, energy and protein, and the associated losses and loss rates, through processes within the global food system, in 2011.

Type	Total	Harvested	Food	Processing	Feed	Forage Crops	Seed	Animal products	Net stock variation	Non-food use	Losses	Rate of loss
Cropland NPP												
Dry mass (Gt)	16.0	4.33									11.67	73.0%
Energy (EJ)	192	64.7									127	66.3%
Protein (Mt)	1600	502									1098	68.7%
Wet mass (Gt)	45.71	9.76									35.96	78.7%

Grassland NPP												
Dry mass (Gt)	11.8	2.48									9.32	78.9%
Energy (EJ)	118	24.8									93	78.9%
Protein (Mt)	826	174									652	78.9%
Wet mass (Gt)	59.00	12.42									46.58	78.9%

Crops harvested [Total is the quantity of primary crops harvested. Food is the quantity of primary crops delivered to consumers.]												
Dry mass (Gt)	4.33		1.33	0.91	0.82	0.44	0.08		0.03	0.29	0.43	10.0%
Energy (EJ)	64.7		19.4	15.2	11.8	5.2	1.2		0.4	4.1	7.3	11.3%
Protein (Mt)	518		137	200	78	26	10		3	26	38	7.6%
Wet mass (Gt)	9.76		3.19	2.36	1.16	1.44	0.14		0.03	0.61	0.82	8.4%

Processed commodities [Total is the quantity of crop processed. Food is the quantity of processed commodities delivered to consumers.]												
Dry mass (Gt)	0.91		0.28		0.25				0.01	0.14	0.22	24.2%
Energy (EJ)	15.2		6.1		2.8				0.2	3.9	2.23	14.7%
Protein (Mt)	200		1		104				− 0	28	67	33.4%
Wet mass (Gt)	2.36		0.51		0.29				0.01	0.15	1.40	59.2%

Livestock production [Total is the inputs (feed and harvested grass), which result in a quantity of edible animal products.]												
Dry mass (Gt)	4.00							0.24			3.76	94.0%
Energy (EJ)	44.9							5.8			39.1	87.2%
Protein (Mt)	387							71			315	81.7%
Wet mass (Gt)	15.40							1.14			11.78	92.6%

Animal products [Total is the production of edible animal products. Food is the quantity delivered to consumers. Feed includes eggs hatched for poultry]												
Dry mass (Gt)	0.24		0.21		0.01				0.00	0.01	0.01	2.0%
Energy (EJ)	5.8		5.0		0.3				0.0	0.4	0.1	1.9%
Protein (Mt)	71		65		3				− 0	1	2	2.3%
Wet mass (Gt)	1.14		1.00		0.09				− 0.00	0.03	0.03	2.6%

Food consumption [Total is the food reaching consumers. Food is the quantity consumed]												
Dry mass (Gt)	1.82		1.66								0.16	9.0%
Energy (EJ)	30.6		28.0								2.6	8.6%
Protein (Mt)	203		185								18	9.0%
Wet mass (Gt)	4.70		4.22								0.48	10.1%

Food requirements [Total is the food consumed. Food is the quantity required for human population, with dry and wet mass using the energy over-consumption ratio.]												
Dry mass (Gt)	1.66		1.49								0.17	10.3%
Energy (EJ)	28.0		25.1								2.9	10.3%
Protein (Mt)	185		133								51	27.9%
Wet mass (Gt)	4.22		3.79								0.43	10.3%

**Fig. 2:**
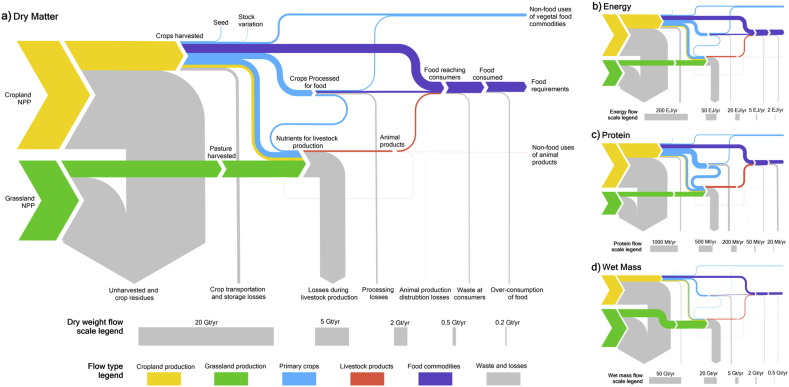
Main flows in the global food system in 2011 from plant growth to human consumption, in: a) dry matter, b) energy, c) protein mass, and d) wet mass. Arrows denote the transfer from one process to another, and their width is proportional to the amount of mass or energy per year. Two flows are shown from harvested crops to livestock production, one for primary food crops (light blue) another for forage crops (yellow). The aggregate size of the cropland and grassland net primary production (NPP) flows are displayed as equivalent sizes across the four panels. The loss and waste flows include a substantial proportion of unharvested biomass and manure that will break down in the soil, providing nutrients for subsequent production.

Fig. 2 as Sankey diagrams in which the size of a flow is indicated by the width of a line (Schmidt, 2008, The Economist, 2011).

The results show the small fraction of total agricultural NPP that is consumed as food. The mass, energy or protein needed to meet global human nutritional requirements as a percentage of total net production in cropland and grassland varies from 3.6–8.1%, depending on whether calculated in mass, energy or protein terms, or 4.0–9.0% for the food eaten, with the lowest rate for wet mass and highest for energy (Table 2). The absolute overall system losses are dominated by agricultural residues and other losses prior to harvest (both of cropland and grassland), with losses of 66–79% that account for around 80% of all losses (Table 1). However, the highest loss rate for the stages considered occurs for livestock production, with losses of 81–94% (Table 1). These high loss rates for livestock production do not result in greater absolute losses as the inputs to livestock production are smaller because they include the losses prior to crop and grassland harvesting, and because not all biomass harvested is used for livestock production.

**Table 2:** Percentage rates between stages of the food system.

Source	Destination	Dry matter (%)	Energy (%)	Protein (%)	Wet mass (%)
Net primary production from cropland and grassland	Food required	5.3	8.1	5.5	3.6
	Food consumed	6.0	9.0	7.6	4.0
	Food reaching consumers	6.5	9.9	8.4	4.5
	Non-food uses	1.6	2.7	2.3	0.8
	Losses (excluding over-consumption)	92.4	88.3	90.1	95.2
	Losses (including over-consumption)	93.0	89.2	92.3	95.6

Harvested crops and grassland^a^	Food required	22.2	28.6	20.1	17.2
	Food consumed	24.8	31.9	27.8	19.2
	Food reaching consumers	27.2	34.9	30.6	21.4
	Non-food uses	6.7	9.5	8.3	3.6
	Losses (excluding over-consumption)	68.5	58.6	63.9	77.2
	Losses (including over-consumption)	71.0	61.8	72.4	79.2

Harvested crops^a^ (not including harvested grassland and forage crops)	Food required	39.5	43.6	27.8	46.8
	Food consumed	44.0	48.6	38.5	52.2
	Food reaching consumers	48.4	53.2	42.3	58.1
	Non-food uses	12.0	14.5	11.4	9.8
	Losses (excluding over-consumption)	44.0	36.9	50.1	38.1
	Losses (including over-consumption)	48.5	41.8	60.8	43.4

Note:

a Stock variation and uses for seed are accounted for by subtracting them from the harvested crop values prior to calculating rates.

### 3.1 Post-crop harvest

The losses after harvest are also substantial. Only 19.2–31.9% - less than a third - of biomass harvested from crops or grass is finally consumed by humans (Table 2), with an additional 3.6–9.5% used for non-food uses. If the biomass harvested from grassland and forage crops are disregarded the rates rise to 42.3–58.1% of harvested crop biomass being consumed as food, and an additional 9.8–14.5% with non-food uses, giving a loss rate of 36.9–50.1% (Table 2). If consumption in excess of nutritional requirements is included as a loss, the total loss rate rises to 41.8–60.8%.

**Fig. 3:**
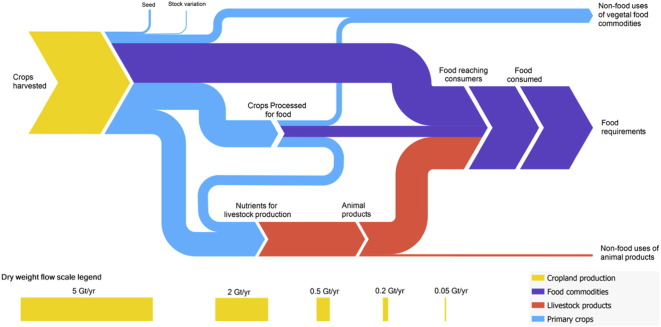
Embodied harvested crops (without forage crops) through stages in food system in dry matter terms.

**Fig. 4:**
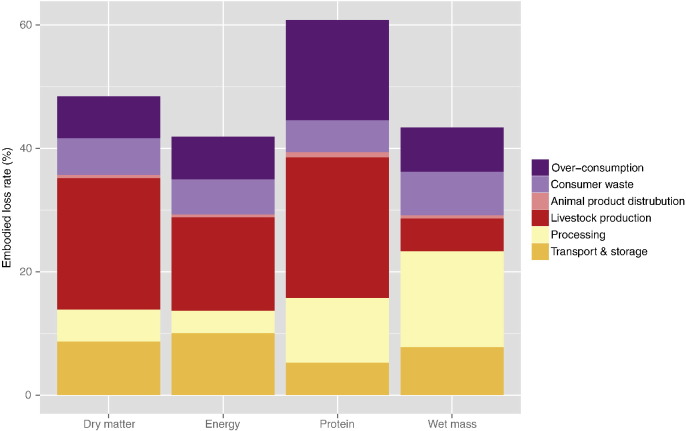
Losses of harvested crops (excluding grassland and forage crop inputs to livestock production) by stage in the food system, using embodied loss rates.

The percentage of loss at each stage (Table 1) allows fair comparison of the rates of losses between stages, but does not put them into the context of the whole system, as not all biomass goes through all stages (e.g. livestock production). Calculating the percentage of overall loss that occurs at each stage shows the losses in a system-wide context, but loss rates at later stages are biased towards smaller percentages as the total quantities at these subsequent stages are lower; i.e. no account is taken of the compounding of losses from proceeding stages (e.g. Fig. S1). Therefore, the embodied quantities were used (e.g. Fig. 3) to calculate the losses of harvested crops associated with each stage (Fig. 4 and Table S5).

The largest losses of dry matter, energy and protein occur in livestock production, but most wet mass is lost during processing. When considering only feed used by livestock, i.e. ignoring livestock inputs from grassland or forage crops, livestock production accounts for 40.4–60.8% of all losses from crop harvest to food consumption. For example, in dry matter terms, 1.06 Gt of feed from crops (plus 0.44 Gt of forage crops and 2.48 Gt of grass) are consumed by livestock to produce 0.24 Gt of animal products. Just considering the feed inputs of food crops the associated loss is 0.82 Gt, or 46.1% of all losses between harvest and food consumption. If adjusted for cumulative embodied losses, this falls slightly to 43.9%. Animal feeds are relatively dry (with a DM content of 74%, compared to a mean of 44% for primary crops), and animal products relatively wet (21%), and therefore the livestock production losses appear smaller for wet mass (1.44 Gt of feed used to produce 1.14 Gt of animal products).

### 3.2 Processing losses

Losses during processing are considerable (15–59% of crops processed), but vary greatly between dry matter, energy, protein and wet mass (Table 1 and Fig. 4). The reason for this variation can be seen by looking at sugar cane and sugar beet. Sugar cane represents the single largest primary crop processed, with 1271 Mt of sugar cane processed globally, in 2011. This sugar cane and 247 Mt of sugar beet produced 170 Mt of raw sugar, 9 Mt of non-centrifugal sugar and 56 Mt of molasses (FAOSTAT, 2015a), implying a processing loss of 1280 Mt or 84%. Sugar cane and beet processing are considered together as the FAOSTAT (2015a) data provide the total sugar produced, but not the quantity produced from each source. Most of the sugar processing losses are in the form of water, as sugar cane and beet have high moisture contents and the sugar has no water content, with 344 Mt of dry matter being processed into 222 Mt of sugar product, giving a substantially smaller loss rate of 35%. Furthermore, processed sugar products are high in energy and therefore the losses in energy are smaller than dry matter (22%). However, sugar contains no protein (although the molasses and non-centrifugal sugar do contain some protein) and so loss rates are high in terms of protein (92%). The main sugar cane by-products are cane tops and bagasses (the fibrous residue after processing of the sugar cane) (Paturau, 1987). Bagasses (with a 50% moisture content) accounts for around 30% of sugar cane processed and is often used as a primary fuel source for the sugar mills (Hofsetz and Silva, 2012). The use of bagasses as a source of bioenergy was not included in the results presented here.

### 3.3 Stock variation

The results show low levels of net stock variation (< 1% of production, Table 1), but with some differences in sign between dry, wet, energy or protein terms. This occurs as commodities that are increasing or decreasing in stock levels are both included, with positive values indicating commodities used to supply stocks, and negative values commodities taken from stocks. For example, if a relatively high protein density commodity was supplied from stocks when a somewhat larger mass of a lower protein density commodity was adding to stocks, this would lead to a positive net stock variation in mass and a negative one for protein.

## 4 Discussion

### 4.1 Comparison to other food loss and waste studies

Previous studies have found that approximately one third of food (in wet mass) is lost from harvest to consumption, including losses during harvesting and consumption (Gustavsson et al., 2011), without accounting for losses in livestock production. This study includes these losses. Furthermore, although harvest losses are included within the wider scope of agricultural production losses calculated here, they are not separately quantified, due to lack of suitable data. This differs from the approach of Gustavsson et al., 2011. Such differences make direct comparisons to previous studies difficult. The closest comparison that can be made to Gustavsson et al., (2011) is between the embodied loss rates from crops harvested to food eaten, excluding livestock production, which suggest that 31% wet mass of crops is lost (or 20% of dry matter), and the 33% overall losses from Gustavsson et al., (2011). Kummu et al. (2012) followed a similar method to Gustavsson et al., (2011), finding a loss of 24% in energy terms, while the approximately equivalent result here is for a 20% energy loss (22% in protein). Cassidy et al. (2013) calculated at only 12% of energy in crops feed to livestock are consumed in the human diet. The 88% loss of calories in livestock production equates almost exactly to the 87.2% loss found here (Table 1). Comparison with these previous studies suggests that the loss rates found here are broadly similar over a range of losses.

### 4.2 Suitability of wet mass to measure losses

Using wet mass to quantify losses is a prevalent approach in previous studies of food losses and waste (Gustavsson et al., 2011, Parfitt et al., 2010), but is potentially misleading. First, aggregating wet mass values for dissimilar products has the potential to introduce unintended effects (Alexandratos and Bruinsma, 2012). For example, if losses from high moisture content foods with higher rates of loss (e.g. soft fruits and vegetables) are aggregated with drier commodities with lower rates of loss (e.g. cereals), the resultant overall loss will be higher in wet mass terms than if calculated as dry matter. The differences based on the terms used may lead to erroneous inferences about the overall rates of losses. Second, changes in moisture content during processing will influence the calculated losses if this water content is included. The results suggest that processing of primary crops is associated with a substantial net loss of water, which is reflected in the wet mass losses. However, it is likely that the losses of energy and nutrients are of greater importance and relevance than the rate of water loss (or addition) that occurs during processing. Therefore, when aggregating dissimilar products or considering processing of products, wet mass should be used with caution, and other terms may be preferable.

### 4.3 Agricultural production efficiencies

The results demonstrate that agricultural production inefficiencies (in both crop and livestock) are the dominant contributions to the overall losses within the food system, when considering either harvested crops or all biomass (Table 1 and Fig. 2). Harvested crops and grass are influenced by agricultural practices and plant breeding. Both the total rate of primary production and also the percentage that is harvested have been increasing over time, in large part due to increasing crop yields (Krausmann et al., 2013). Livestock production efficiencies have also been increasing over time (Havenstein, 2006), but still are responsible a substantial loss. The extent to which climate change, plant and animal breeding, and agricultural practices and technologies will develop and interact in future is clearly relevant (Engström et al., 2016, Garnett et al., 2013, Godfray et al., 2010, Herrero et al., 2016, Jaggard et al., 2010). All influence future production efficiencies (as well as the total agricultural NPP), and therefore overall food system losses.

The uses and losses of harvested crops only were considered in the results (Table 2, Fig. 3, Fig. 4). The contribution of grassland to animal nutrition could be argued to be of less direct conflict with human food production than the use of food commodities for feed (Foley et al., 2011). Grass is not edible by humans, and land used for grazing may be unsuitable for producing other crops and so, may not compete directly with other food production systems (Capper et al., 2013). The results that do not include any contribution from grassland and forage crops implicitly assume that livestock production does not compete with the production of food from cropland, except through the use of feed. However, not all grassland is unsuitable for other agricultural uses, and pasture has been expanding more rapidly than cropland over the past 50 years (Alexander et al., 2015), implying that this assumption is only partially valid. Therefore, livestock production losses that only consider crop use understate the impact on the agricultural system as a whole. Despite this moderate approach to livestock production, the associated inputs and losses are substantial. The proportion of harvested crops used for livestock varies from 28% for wet mass (in line with previous values (Foley et al., 2011)) to 57% for protein, with 40% for dry matter and 36% for energy. That is, the proportion of harvested crop used for feed is lowest in wet mass (the terms typically used, but that is potentially misleading, as discussed above). Furthermore, the highest losses from any stage (other than for wet mass) are associated with livestock production (Fig. 4). Livestock production therefore represents a major source of losses often not included in studies of losses and waste in the food system (Gustavsson et al., 2011, Kummu et al., 2012), and this difference in method contributes to the higher overall loss rates found here.

### 4.4 Uncertainties in the analysis

There are few estimates of global NPPs by land cover type, compared to studies providing the total NPP. Here we use the figures from Ito and Oikawa (2004) of 8.0 PgC/yr and 5.9 PgC/yr for cropland and grassland respectively (Table S1), while Chen et al. (2014) finds 11.05 PgC/yr and 5.5 PgC/yr, and the human appropriation of net primary production (HANPP) values at 2005 from Krausmann et al. (2013) are 7.5 PgC/yr and 4.5 PgC/yr respectively. In comparison to these, Field et al. (2008) found somewhat lower cropland 6.8 PgC/yr NPP, with a higher grassland NPP 11.6 PgC/yr, perhaps arising due to the definitional issues for grassland (Alexander et al., 2016b, Prestele et al., 2016). Additionally, agricultural NPP figures change over time as agricultural areas and practices alter, therefore the inconsistency between the 2004 NPP estimates and 2011 FAO data may lead to an underestimate of the harvest losses, particularly for croplands. Translating the NPPs in terms other than dry matter creates additional uncertainty, as they involve global average energy, protein and moisture contents. Although the NPP values must be viewed with caution, such uncertainty only impacts a limited set of the results of this analysis. The NPP values do not impact the quantities calculated at subsequent stages, as these are derived from the FAO data and human nutritional requirements (Fig. 1), and consequently the NPP values have no impact on the losses between processes at these later stages (e.g. losses of harvested crops, Table 2 and Fig. 3). Additionally, the FAO data used in the analysis has a level of uncertainty that is difficult to determine; as it is based on global panel data it is inherently of varying quality. However, the FAO compiled data used is the best available source of such global data, and as such has previously been widely used for academic and other purposes. Validation checks were also run to ensure internal consistency of input data and consistency with the results, e.g. that all quantities are conserved.

Livestock feed inputs may be understated as some sources of feeds from food residues, and by-products from other agricultural processing are not included. The majority of these agricultural residues are straw (including stover from coarse grains), with around 4 Gt DM globally, but low digestibility and voluntary intake has limited their feed use (Mahesh and Mohini, 2014, Sarnklong et al., 2010), and with rates of use in decline (FAO, 2006). Not including these feeds will reduce the estimate of biomass provided to livestock from cropland. As the animal product quantities produced are derived separately from data in FAOSTAT (2015b), lower feed inputs will result in lower loss rates being calculated for livestock production (e.g. in Fig. 4 and Table 1). Global average feed conversion ratios have been used to estimate the livestock feed requirements, however these are uncertain and vary with intensity of production, animal breeding and management practices (Alexander et al., 2015, Fairlie, 2010, Smil, 2002). Any inaccuracies in feed conversion ratios would create a shift between losses in grassland harvest and livestock production, but not change to other system losses. For example, low feed conversion ratios would less feed being estimated for livestock production, which would cause higher unharvested grassland losses but an offsetting reduction in animal production losses. The livestock production losses include manure, methane and nitrous oxides emissions, metabolised energy, and carcass materials. However, some of the animal by-products find a range of uses, e.g. leather and gelatine, as well as also creating issues for disposal (Jayathilakan et al., 2012). Any beneficial uses of animal by-products are not captured by the analysis here, which therefore understates the non-food uses of these products.

The inequalities in food distribution both within and between countries (Porkka et al., 2013), may have led to under-estimating the food system losses due to consumption in excess of nutrient requirements. Globally, 37% of men and 38% of women were overweight in 2014 (Ng et al., 2014), while approximately 12% of people were undernourished between 2010 and 2012 (FAO et al., 2015). As the analysis conducted here is done at the global level, it averages out the wide range of nutritional consumptions between individuals. Therefore, the losses associated with over eating will be biased towards being too low, as the over-consumption of food is partially offset by people who are under-nourished.

## 5 Conclusions

Both consumer behaviour and production practices play crucial roles in the efficiency of the food system. This study considers the interconnectedness of the food system and the losses occurring, using primarily empirical data. The results emphasise the substantial losses occurring during livestock production, and reveals the magnitude of losses from consumption of food in excess of human nutritional requirements. The greatest rates of loss were associated with livestock production, and consequently changes in the levels of meat, dairy and egg consumption can substantially affect the overall efficiency of the food system, and associated environmental impacts (e.g. greenhouse gas emissions) (Lamb et al., 2016). It is therefore regrettable from environmental and food security perspectives that rates of meat and dairy consumption are expected to continue to increase as average incomes rise (Kearney, 2010, Keyzer et al., 2005, McMichael et al., 2007), potentially lowering efficiency of the overall food system, as well as increasing associated negative health implications (e.g. diabetes and heart disease) (Hu, 2011, Tilman and Clark, 2014). Changes in livestock production practices and animal genetics may increase efficiencies to offset some of these effects (Havlík et al., 2014, Le Cotty and Dorin, 2012), but may be insufficient to do so completely.

The effect of changes in consumer behaviour has received substantial research focus, e.g. the role of diet and dietary changes in agricultural resource use and environmental sustainability (Bajželj et al., 2014, Smith et al., 2013, Stehfest et al., 2009, West et al., 2014, Wirsenius et al., 2010). Furthermore, the links between diet, obesity and human health have been widely recognised (NCD Risk Factor Collaboration, 2016, Wang and Beydoun, 2009). However, until recently, less attention appears to have been given to the sustainability implications of over-consumption (Springmann et al., 2016). The results here suggest that system losses from over-consumption of food are at least as substantial as the losses from food discarded by consumers (Fig. 4), and therefore have comparable food security and sustainability implications. Consequently, greater research focus may be required to better understand causes, effects and solutions for over-consumption. Changes to influence consumer behaviour, e.g. eating less animal products, reducing food waste, and lowering per capita consumption to be closer to nutrient requirements will all help to provide the rising global population with food security in a sustainable manner.

## Appendix A Supplementary information

Supplementary information. Image 1
