# Supplementary material for: Losses, inefficiencies and waste in the global food system
Source: Agric Syst. 2017 May;153:190–200. doi: 10.1016/j.agsy.2017.01.014 (PMC5437836; doi:10.1016/j.agsy.2017.01.014)
Supplement: Supplementary file 1 — Supplementary information. [file mmc1.pdf]

Supplementary Information: Losses, inefficiencies and waste in the global food system

SI Methods

The R source code for the analysis can be obtained from https://bitbucket.org/alexanpe/foodwaste, and the data analysed can be downloaded from http://faostat3.fao.org.

Table S1. Global NPPs in mass (both dry matter (DM) and wet), energy and protein terms for cropland and grassland.

Land cover type	Total NPP *[Source: Ito & Oikawa, 2004]* (PgC/yr)	Total NPP (Gt DM/yr)	Energy content (MJ/kg DM)	Total Energy (EJ/yr)	Protein content (%)	Total protein (Mt/yr)	Dry matter (%)	Total Wet NPP (Gt/yr)
Cropland	8.0	16.0	12	192	10	1600	35	45.7
Grassland	5.9	11.8	10	118	7	826	20	59

Table S2. Commodities considered in the analysis, including the type of commodity (primary crop, processed commodity or animal product), the energy, protein and dry matter contents, and the commodity group used to estimate consumer waste.

Item	Commodity type	Energy (MJ/kg)	Protein (g/kg)	Dry Matter	Consumer waste commodity group
Animal fats	Animal product	28.6	9	100.0%	Meat
Apples	Primary crop	1.8	2	14.4%	Fruits and vegetable
Bananas	Primary crop	2.6	8	25.1%	Fruits and vegetable
Barley	Primary crop	11.1	76	88.0%	Cereals
Beans	Primary crop	14.5	221	88.3%	Oilseeds and pulses
Beer	Processed commodity	1.9	4	8.0%	Cereals
Beverages Alcoholic	Processed commodity	12.3	0	13.4%	Cereals
Beverages Fermented	Processed commodity	1.7	4	36.1%	Cereals
Bovine Meat	Animal product	6.7	142	32.9%	Meat
Cassava	Primary crop	4.0	6	40.3%	Roots and tubers
Cereals Other	Primary crop	13.8	85	88.0%	Cereals
Citrus Other	Primary crop	1.1	5	17.0%	Fruits and vegetable
Cocoa Beans	Primary crop	11.8	73	88.0%	Oilseeds and pulses
Coconut Oil	Processed commodity	37.2	0	100.0%	Oilseeds and pulses
Coconuts - Incl Copra	Primary crop	5.8	15	47.5%	Fruits and vegetable
Coffee	Primary crop	1.3	58	88.0%	Oilseeds and pulses
Copra Cake	Processed commodity	11.3	415	93.0%	Oilseeds and pulses
Cotton lint	Processed commodity	20.0	900	97.0%	Fibre
Cottonseed	Processed commodity	18.0	310	88.0%	Oilseeds and pulses
Cottonseed Cake	Processed commodity	11.3	415	92.2%	Oilseeds and pulses
Cottonseed Oil	Processed commodity	38.7	0	100.0%	Oilseeds and pulses
Dates	Primary crop	7.0	17	79.5%	Fruits and vegetable
Eggs	Animal product	6.1	114	23.9%	Meat
Fish Seafood	Primary crop	2.8	103	20.0%	Fish and seafood
Forage crops	Primary crop	3.6	18	30.0%	Forage
Fruits Other	Primary crop	1.8	5	15.0%	Fruits and vegetable
Grapefruit	Primary crop	1.4	3	10.4%	Fruits and vegetable
Grapes	Primary crop	2.5	6	19.5%	Fruits and vegetable
Groundnut Cake	Processed commodity	11.3	415	89.3%	Oilseeds and pulses
Groundnut Oil	Processed commodity	38.3	0	100.0%	Oilseeds and pulses
Groundnuts	Primary crop	23.0	239	90.0%	Oilseeds and pulses
Lemons Limes	Primary crop	0.8	4	14.7%	Fruits and vegetable
Maize	Primary crop	12.9	75	86.3%	Cereals

Maize Germ Oil	Processed commodity	39.6	0	100.0%	Oilseeds and pulses
Meat Other	Animal product	4.8	182	33.0%	Meat
Milk	Animal product	2.4	34	11.9%	Milk
Millet	Primary crop	12.6	81	90.2%	Cereals
Molasses	Processed commodity	9.5	40	73.0%	Sugar and sweeteners
Mutton & Goat Meat	Animal product	9.2	141	40.5%	Meat
Nuts	Primary crop	10.7	66	90.0%	Oilseeds and pulses
Oats	Primary crop	8.0	82	87.9%	Cereals
Oil palm fruit	Primary crop	10.0	150	34.0%	Oilseeds and pulses
Oilcrops Oil Other	Processed commodity	30.4	0	100.0%	Oilseeds and pulses
Oilcrops Other	Primary crop	11.1	212	88.0%	Oilseeds and pulses
Oilseed Cakes Other	Processed commodity	11.3	415	90.0%	Oilseeds and pulses
Olive Oil	Processed commodity	37.3	0	100.0%	Oilseeds and pulses
Olives	Primary crop	4.1	10	24.7%	Oilseeds and pulses
Onions	Primary crop	1.7	14	10.9%	Fruits and vegetable
Oranges Mandarines	Primary crop	1.2	5	17.7%	Fruits and vegetable
Palm Oil	Processed commodity	37.3	2	100.0%	Oilseeds and pulses
Palmkernel Cake	Processed commodity	11.3	415	91.2%	Oilseeds and pulses
Palmkernel Oil	Processed commodity	35.0	0	100.0%	Oilseeds and pulses
Peas	Primary crop	15.2	228	91.4%	Oilseeds and pulses
Pigmeat	Animal product	12.1	108	38.9%	Meat
Pimento	Primary crop	14.0	126	6.9%	Fruits and vegetable
Pineapples	Primary crop	1.6	3	14.0%	Fruits and vegetable
Plantains	Primary crop	3.6	7	34.7%	Fruits and vegetable
Potatoes	Primary crop	2.9	16	21.0%	Roots and tubers
Poultry Meat	Animal product	6.1	128	26.8%	Meat
Pulses Other	Primary crop	14.8	218	88.0%	Oilseeds and pulses
Rape and Mustard Cake	Processed commodity	10.5	351	90.1%	Oilseeds and pulses
Rape and Mustard Oil	Processed commodity	37.6	0	100.0%	Oilseeds and pulses
Rape and Mustardseed	Primary crop	22.0	205	91.7%	Oilseeds and pulses
Rice	Primary crop	15.8	71	88.6%	Cereals
Ricebran Oil	Processed commodity	38.0	0	100.0%	Oilseeds and pulses
Roots Other	Primary crop	4.9	16	20.0%	Roots and tubers
Rye	Primary crop	11.3	76	86.8%	Cereals
Seed cotton	Primary crop	19.0	510	90.0%	Oilseeds and pulses
Sesame seed	Primary crop	21.9	175	97.2%	Oilseeds and pulses
Sesameseed Cake	Processed commodity	11.3	415	88.0%	Oilseeds and pulses
Sesameseed Oil	Processed commodity	41.5	0	100.0%	Oilseeds and pulses
Sorghum	Primary crop	13.2	92	87.4%	Cereals
Soyabean Cake	Processed commodity	8.2	385	87.9%	Oilseeds and pulses
Soyabean Oil	Processed commodity	36.9	1	100.0%	Oilseeds and pulses
Soyabeans	Primary crop	14.8	332	91.5%	Oilseeds and pulses
Spices Other	Primary crop	14.4	113	88.0%	Oilseeds and pulses
Sugar	Processed commodity	14.6	0	100.0%	Sugar and sweeteners
Sugar beet	Primary crop	3.0	15	18.8%	Roots and tubers
Sugar cane	Primary crop	2.6	22	23.4%	Fruits and vegetable
Sugar non-centrifugal	Processed commodity	14.1	8	100.0%	Sugar and sweeteners
Sunflower seed	Primary crop	15.0	108	92.8%	Oilseeds and pulses
Sunflowerseed Cake	Processed commodity	11.3	415	89.0%	Oilseeds and pulses
Sunflowerseed Oil	Processed commodity	37.4	0	100.0%	Oilseeds and pulses
Sweet potatoes	Primary crop	4.1	11	21.0%	Roots and tubers
Sweeteners Other	Processed commodity	11.9	1	100.0%	Sugar and sweeteners
Tea	Primary crop	2.0	100	95.0%	Oilseeds and pulses
Tomatoes	Primary crop	0.8	10	5.5%	Fruits and vegetable
Vegetables Other	Primary crop	1.1	14	10.0%	Fruits and vegetable
Wheat	Primary crop	12.6	91	87.0%	Cereals
Wine	Processed commodity	2.4	0	13.4%	Fruits and vegetable
Yams	Primary crop	4.4	17	30.4%	Roots and tubers

Table S3. Countries included in each world regions for consumer waste calculations.

**Europe including Russia**				
Albania	Cyprus	Hungary	Netherlands	Slovenia
Armenia	Czech Republic	Iceland	Norway	Spain
Austria	Denmark	Ireland	Poland	Sweden
Azerbaijan	Estonia	Italy	Portugal	Switzerland
Belarus	Finland	Latvia	Republic of Moldova	Macedonia
Belgium	France	Lithuania	Romania	Ukraine
Bosnia & Herzegovina	Georgia	Luxembourg	Russian Federation	United Kingdom
Bulgaria	Germany	Malta	Serbia	
Croatia	Greece	Montenegro	Slovakia	
**North America and Oceania**				
Australia	Fiji	New Caledonia	Samoa	United States of America
Bermuda	French Polynesia	New Zealand	Solomon Islands	Vanuatu
Canada	Kiribati			
**Industrialized Asia**				
China	DPR of Korea	Japan	Republic of Korea	
**Sub-Saharan Africa**				
Angola	Cote d'Ivoire	Guinea-Bissau	Niger	Swaziland
Benin	Djibouti	Kenya	Nigeria	Togo
Botswana	Equatorial Guinea	Lesotho	Rwanda	Uganda
Burkina Faso	Eritrea	Liberia	Sao Tome and Principe	Tanzania
Burundi	Ethiopia	Malawi	Senegal	Zambia
Cameroon	Gabon	Mali	Sierra Leone	Zimbabwe
Central African Rep.	Gambia	Mauritania	Somalia	
Chad	Ghana	Mozambique	South Africa	
Congo	Guinea	Namibia	Sudan	
**North Africa, West and Central Asia**				
Algeria	Israel	Lebanon	Saudi Arabia	Turkmenistan
Brunei Darussalam	Jordan	Libya	Syria	United Arab Emirates
Cabo Verde	Kazakhstan	Mongolia	Tajikistan	Uzbekistan
Egypt	Kuwait	Morocco	Tunisia	Yemen
Iraq	Kyrgyzstan	Oman	Turkey	
**South and Southeast Asia**				
Afghanistan	India	Myanmar	Sri Lanka	Madagascar
Bangladesh	Indonesia	Nepal	Thailand	Maldives
Bhutan	Iran	Pakistan	Viet Nam	Mauritius
Cambodia	Malaysia	Philippines	Lao PDR	Timor-Leste
**Latin America**				
Antigua and Barbuda	Chile	El Salvador	Mexico	St Vincent & Grenadines
Argentina	Colombia	Grenada	Nicaragua	Suriname
Bahamas	Costa Rica	Guatemala	Panama	Trinidad and Tobago
Barbados	Cuba	Guyana	Paraguay	Uruguay
Belize	Dominica	Haiti	Peru	Venezuela
Bolivia	Dominican Republic	Honduras	Saint Kitts and Nevis	
Brazil	Ecuador	Jamaica	Saint Lucia	

Table S4. Assumed percentage rates of waste after reaching consumers by commodity type and region.

	Europe including Russia	North America and Oceania	Industrialized Asia	Sub-Saharan Africa	North Africa, West and Central Asia	South and Southeast Asia	Latin America
Cereals	25	27	20	1	12	3	10
Fish and seafood	11	33	8	2	4	2	4
Fruits and vegetable	19	28	15	5	12	7	10
Meat	11	11	8	2	8	4	6
Milk	7	15	5	0.1	2	1	4
Oilseeds and pulses	4	4	4	1	2	1	2
Roots and tubers	17	30	10	2	6	3	4
Sugar and sweeteners	4	4	4	1	2	1	2

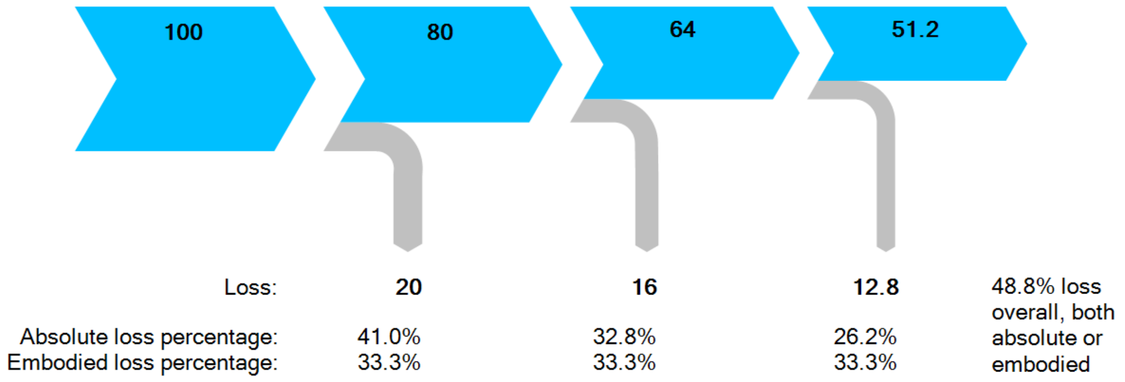

Figure S1. Example of a simple linear flow, with three processes each having 20% loss (quantities in bold), illustrating the need for adjustment to embodied quantities for unbiased comparison of losses between processes.

SI Results

Table S5. Percentage of losses that occur at each stage in the food system, from harvested crops (not including grassland inputs to livestock production and forage crops) to food required for human nutrition. Rates are given as the percentages of the total embodied loss (adjusting for the compounding of losses that have occurred in previous stages).

**Stage in food system**	**Dry Matter (%)**	**Energy (%)**	**Protein (%)**	**Wet mass (%)**
Transportation and storage	18.0	24.0	8.7	17.9
Processing	10.7	8.8	17.2	35.8
Livestock production	43.9	36.1	37.5	12.3
Animal product distribution	1.1	1.1	1.4	1.2
Consumer waste	12.2	13.7	8.6	16.3
Over-consumption	14.0	16.4	26.6	16.5
Total	*100*	*100*	*100*	*100*
